# Understanding the role and deployment of volunteers within specialist palliative care services and organisations as they have adjusted to the COVID-19 pandemic: A multi-national EAPC volunteer taskforce survey

**DOI:** 10.1177/02692163221135349

**Published:** 2023-02

**Authors:** Catherine Walshe, Leszek Pawłowski, Sophie Shedel, Steven Vanderstichelen, Melissa J Bloomer, Anne Goossensen, Joaquín T Limonero, Karen Sangild Stoelen, Chiara Caraffa, Leena Pelttari, Ros Scott

**Affiliations:** 1 International Observatory on End of Life Care, Division of Health Research, Lancaster University, Lancaster, UK; 2 Department of Palliative Medicine, Medical University of Gdańsk, Gdańsk, Poland; 3 Lancaster University, Lancaster, UK; 4 Vrije Universiteit Brussel, Gent, Belgium; 5 Griffith University School of Nursing and Midwifery & Princess Alexandra Hospital Intensive Care Unit, Brisbane, QLD, Australia; 6 University of Humanistic Studies, Utrecht, The Netherlands; 7 Universitat Autònoma de Barcelona, Bellaterra (Cerdanyola del Vallès), Barcelona, Spain; 8 University College Copenhagen, Copenhagen, Denmark; 9 Italian Federation for Palliative Care, Milan, Italy; 10 Hospice Austria, Vienna, Austria; 11 University of Dundee, Dundee, Scotland, UK

**Keywords:** COVID-19, COVID-19 pandemic, hospices, palliative care, volunteers, workforce

## Abstract

**Background:**

Early indications were of a major decline in specialist palliative care volunteer numbers during COVID-19. It is important that ongoing deployment and role of volunteers is understood, given the dependence of many palliative care services on volunteers for quality care provision.

**Aim:**

To understand the roles and deployment of volunteers in specialist palliative care services as they have adjusted to the impact of COVID-19.

**Design:**

Observational multi-national study, using a cross-sectional online survey with closed and free-text option questions. Disseminated via social media, palliative care networks and key collaborators from May to July 2021.

**Setting/participants:**

Any specialist palliative care setting in any country, including hospices, day hospices, hospital based or community teams. The person responsible for managing the deployment of volunteers was invited to complete the survey.

**Results:**

Valid responses were received from 304 organisations (35 countries, 80.3% Europe). Most cared for adults only (60.9%), provided in-patient care (62.2%) and were non-profit (62.5%). 47.0% had cared for people with COVID-19. 47.7% changed the way they deployed volunteers; the mean number of active volunteers dropped from 203 per organisation to 33, and 70.7% reported a decrease in volunteers in direct patient/family facing roles. There was a shift to younger volunteers. 50.6% said this drop impacted care provision, increasing staff workload and pressure, decreasing patient support, and increasing patient isolation and loneliness.

**Conclusion:**

The sustained reduction in volunteer deployment has impacted the provision of specialist palliative care. Urgent consideration must be given to the future of volunteering including virtual modes of delivery, micro-volunteering, and appealing to a younger demographic.

**What is already known?**

- Effective use of volunteers is a possible response to the COVID-19 pandemic
- Many specialist palliative care services depend on volunteers for quality care provision
- At the start of the pandemic, volunteering numbers in specialist palliative care dropped significantly

**What this paper adds?**

- The reduction in volunteer deployment in specialist palliative care has been sustained and is reported to have negatively affected quality of care
- Volunteer training largely shifted to real-time online training and covered COVID-19, infection prevention and use of PPE
- Few specialist palliative care organisations have yet created new volunteer roles or ways of working

**Implications for practice, theory or policy**

- Specialist palliative care organisations need to consider how to create new volunteering opportunities that may attract a younger volunteer demographic
- Ways of harnessing community or social action volunteers to be involved in palliative care volunteering are required
- The potential of virtual or remote volunteering in palliative care have to be further developed in ways that are inclusive and do not promote inequity of opportunity

## Background

The COVID-19 pandemic has demonstrated both the possibilities and challenges of the roles of volunteers. Positively, there has been a pivot in many countries to harness the time and skills of volunteers. Effective use of volunteers was highlighted as a possible response to the pandemic,^1^ with calls for mobilising and training a citizen volunteer workforce that is ready and able to connect with patients in need of basic social support.^2^ Examples include ‘micro-volunteering’ where individuals are connected to those needing help, often via social media or other technologies, with examples in India^3^ and in the UK.^4^ Other initiatives include more formal volunteering roles such as village health volunteers in Thailand.^5^ However, it is also apparent that the shift to COVID-19 focused volunteer roles could crowd out existing volunteering for other causes, as found in China where experienced local volunteers rapidly shifted to support needs arising from COVID-19.^6^ There has been a precipitous decline in volunteering across organisations that traditionally rely on a substantial volunteer contribution. A large Australian survey found that since February 2020, almost two-thirds (65.9%) of volunteers had stopped volunteering as a precaution to minimise COVID-19 transmission, equivalent to 12.2 million hours per week.^7^

In specialist palliative care services, which encompass a range of services provided to people with chronic, life-threatening conditions towards the end of life, volunteers can outnumber paid staff, although data on the number of volunteers across countries can be scant.^8^ A UK survey identified 1.5 volunteers to every paid member of staff,^9^ providing up to 8 h a week of care and support^10^ and Dutch ‘Almost at home homes’ typically have one paid coordinator and 80–100 volunteers.^11^ Volunteers offer stability; a Belgian survey identified that 57% of volunteers had been in their current care organisation for at least 6 years, and 36% for over 10 years.^10^ If there has been a decline in palliative care volunteering that mirrors the more general changes in volunteering during the COVID-19 pandemic, this could have substantial impacts on care provision. Early data indicated that at least in the initial days of the pandemic, specialist palliative care volunteering numbers dropped significantly.^12^ A multi-national survey of specialist palliative care providers found that 78% of organisations that deployed volunteers pre-COVID-19 reported less or much less use of volunteers during the early stages of COVID-19 (data collected April–July 2020).^12^ This reduction in volunteers was felt to protect potentially vulnerable volunteers, with policy changes preventing much volunteer support.

It is important that the ongoing deployment and role of volunteers during the COVID-19 pandemic is understood, especially to know if and how services have changed from their immediate response reported in the earlier stages of the pandemic, and to help develop policy for the future, given the dependence that many specialist palliative care services have on volunteers for quality care provision. The aim of this study therefore is to understand the roles and deployment of volunteers in specialist palliative care services as they have adjusted to the impact of COVID-19 on their organisations a year into the pandemic.

## Methods

### Research questions

Over the course of the COVID-19 pandemic:

- How has the deployment and/or roles of volunteers within specialist palliative care services changed, and what has been the impact of any changes?
- What factors contributed to any changes in the deployment and/or roles of volunteers within specialist palliative care services?
- What have been the challenges and opportunities associated with any changes in the deployment and/or roles of volunteers within specialist palliative care services?

***Design*:** Descriptive, observational multi-national study, with cross-sectional online survey of providers of specialist palliative care services. This survey is reported according to the CHERRIES guidelines for reporting on e-surveys.^13^

***Setting***: Specialist palliative care is traditionally delivered wherever patients and those important to them are cared for, and most settings can have volunteers supporting their work. Specialist palliative care is provided by specialised services for patients with complex problems, often requiring a team approach, combining a multi-professional team with an interdisciplinary mode of work. Team members are highly qualified and should have their main focus of work in palliative care.^14^ Such services can include hospices (voluntary and publicly managed), palliative care units, palliative day care centres, palliative home care teams (providing care within the person’s usual place of residence), and palliative support teams (including within acute hospitals). They are distinct from what is sometimes called generalist palliative care services, which are care services in which palliative care is offered but not the primary goal of care provision.

***Inclusion criteria***: Specialist palliative care services and organisations in any country. As per the setting information above, this included: hospices, day hospices, hospital based palliative care teams/wards, home care/community teams and other services that offer specialist palliative care.

***Exclusion criteria*:** No volunteer provision within the service.

***Participants*:** The person responsible for managing the deployment of volunteers within a participating specialist palliative care service, typically the volunteer lead or manager, was invited to complete the online survey on behalf of the organisation. This could include a paid staff member or volunteer with this responsibility.

***Sample***: This survey used a convenience sampling approach, driven by the open method of recruitment such that anyone with access to the link was able to participate, if they met the inclusion criteria. We anticipated a response of between 50 and 300 services, depending on the eventual breadth of the dissemination of the online survey link, estimated from an earlier general palliative care survey,^12^ but the numbers were not restricted or capped.

***Recruitment***: Information about the survey, including the link to access the survey, was openly and widely disseminated through authors’ institutional websites, personal networks and contacts with national palliative care networks and organisations, social media (via advertising through posts on Twitter, Facebook and LinkedIn), and working with the European Association for Palliative Care (EAPC) (e.g. a blog was published inviting eligible organisations to complete the survey). No incentives to complete were offered. All dissemination modes included a link to the online survey, and an invitation to circulate the survey link to others. Potential participants answered screening questions at the start of the survey to confirm eligibility, and clicking to progress to the survey indicated consent.

***Data collection***: The open online survey was built using Qualtrics^XM^ software,^15^ and the full survey is included in Supplemental Materials (S1). Data on key service related information was collected with a suite of questions capturing the deployment of volunteers pre-COVID-19, through the COVID-19 pandemic, and future plans. Both closed and free-text questions were used, together with skip options dependent on given answers; 83 possible questions were asked across 9 blocks. Participants could navigate through the survey using forward and back buttons. The survey was developed by members of the EAPC volunteer taskforce, incorporating some core questions from a previous survey of the impact of COVID-19 on palliative care.^16^ Pilot testing of question wording, format and technical completion was done via EAPC volunteer taskforce members, who asked eligible colleagues to test the survey and link and provide feedback as a check on face validity. The survey was only available in an English language version, although some recruitment materials were translated to national languages. Participants could only complete the survey once, with an automatic reminder prompt 1 week following commencement of the survey if it were not yet complete. Respondents did not receive information about whether they had fully completed the survey. The survey was open from 19.5.2021 to 5.7.2021.

***Data analysis***: Data were downloaded from Qualtrics^QM^ to Microsoft Excel, hosted on Lancaster University secure OneDrive, checked and cleaned to check for potential duplicate entries (using IP, email address or organisation name to ensure only one entry per organisation), and to remove incomplete entries. Entries were judged as sufficiently complete to include in analysis when descriptive organisational information was present, even if answers to all available questions had not been given. There were no completeness checks for participants prior to submission, and no response items that were mandatory or enforced. Pseudonymised quantitative data were transferred to Statistica v13™ (TIBCO Software Inc., Palo Alto, CA, USA). Descriptive analysis of data (e.g. organisational characteristics, volunteer deployment) included the use of frequency counts (including missing data), percentages, measures of central tendency and range. Where data permitted, contingency tables were created using chi-squared tests to compare responses by characteristics considered to potentially have an impact on volunteer deployment (e.g. geography or COVID-19 experience).

For the analysis of free-text comments, data were extracted into Microsoft Excel. Comments tended to be brief, expanding on answers to closed questions.^17,18^ After initial familiarisation, a coding framework was inductively developed through close reading of the text and the use of broad codes to categorise the data, agreed and then applied to the free text data (by RS, CW) using a conventional content analysis technique.^19^ Coding and subsequent higher order categorisation were inductively driven by the content of the free-text comments, with categories identified initially within, and then compared across, the sets of answers to each question.

***Ethics*:** Approval was granted by the Lancaster University Faculty of Health and Medicine Research Ethics Committee (FHMREC20131 18.5.2021).

## Results

The survey received 754 visitors, of whom 17 declared they did not meet the inclusion criteria, 281 provided no data, and 152 did not proceed beyond the screening questions. Valid responses were received from 304 organisations (40.3% of visitors). Of the 304 responses included in the analysis 210 (69.0%) had completed the entire survey. The mean survey progress across all included respondents was 81.5%. Valid responses were received across 35 countries, categorised into geographical regions for analysis (full list of responding countries in Supplemental Materials S2). Descriptive data from these respondents are found in Table 1. Most responding organisations primarily cared for adults (60.9%), were based in Europe (80.3%), and commonly provided in-patient palliative care (62.2%) and/or specialist palliative care home care consulting services (57.6%). Most were charitably funded or non-profit (62.5%).

**Table 1.** Characteristics of responding organisations.

	Number of responses	Percentage
	(*N* = 304)	
Population served by the responding organisation		
Adult patients only	185	60.90
Child patients only	12	3.90
Both adult and child patients served	105	34.50
Missing	2	0.70
Geographical Region of responding organisation		
Western Europe	113	37.20
Northern Europe	17	5.60
Eastern Europe	15	4.90
Southern Europe	49	16.10
British Isles	50	16.50
Asia	15	4.90
Australasia	14	4.60
North America	24	7.90
South America	5	1.60
Africa	2	0.70
Settings in which care offered by each organisation^a^		
In-patient hospice/ward/palliative care unit	189	62.20
Palliative day care centres/services	71	23.40
Hospital palliative care advisory team	84	27.60
Specialist palliative home care service (supporting or consulting about patients at home and/or in the community)	175	57.60
Providing hands on nursing care at home/in the community (e.g. hospice@home, pall@home)	95	31.50
Bereavement services offered	125	41.10
Service management		
Charitable/non-profit	190	62.50
Public	44	14.50
Private	18	5.90
Mixed funding	36	11.80
Missing	16	5.30

a Does not total 100% as organisations could offer multiple services.

Findings are presented taking account of the main areas of the survey and the categorisation and analysis of the free-text comments to illuminate and expand upon these areas. The areas presented are: exposure to COVID-19; changes in volunteer deployment; changes in volunteer training; new or changed volunteer roles; and impact of reduced volunteering.

## Exposure to COVID-19

**Figure 1.**
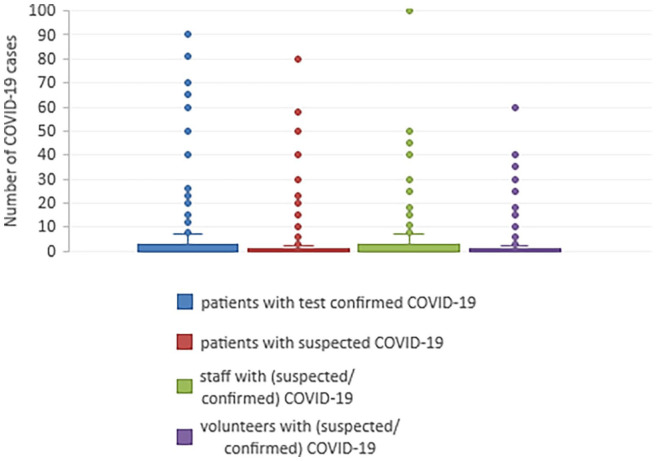
Box and whisker plot of the distribution of cases of COVID-19 experienced by organisations, where the number of cases is ⩽100.

Organisations had different degrees of experience with COVID-19. Their amount of exposure through caring for people with COVID-19, and if their staff or volunteers had COVID-19 is detailed in Table 2, and displayed graphically in Figure 1.

**Table 2.** The experience of organisations to date with COVID-19 since January 2020.

All data are since January 2020	Organisations who had cared for patients with confirmed (by test) cases of COVID-19	Organisations who had cared for patients with suspected (untested but with clinical diagnosis/symptoms) COVID-19	Organisations who had staff with suspected/confirmed COVID-19	Organisations who had volunteers with suspected/confirmed COVID-19
Yes (*n* of organisations and %)	143 (47.0%)	115 (37.8%)	179 (55.9%)	113 (37.3%)
No (*n* of organisations and %)	144 (47.4%)	156 (51.3%)	98(32.2%)	145 (47.7%)
If yes, mean number of cases	350.5	856.9	74.5	6.2
If yes, median number of cases	5	10	4	3
If yes, range of number of cases	0–20,000^a^	0–60,000^a^	0–8000	0–60

a The larger numbers in the range were very few or one organisation likely covering a large area, in countries with very high numbers of those with COVID-19. These had an effect of skewing the mean, but are included for context.

There were few concerns that volunteers had been exposed to or infected with COVID-19 because of their deployment within the organisation. Where volunteers had reported infection, it was usually due to general community transmission:

*Only a limited number of volunteers were in the hospice building from summer 2020. They were tested regularly, along with all people working at or visiting us. No volunteers who supported in the building had suspected COVID-19. Volunteers who did have COVID-19, were those who were either volunteering remotely, or their volunteering was paused. (Respondent 219, UK, Children’s service, multiple settings)*

## Changes in volunteer deployment

Whilst the median number of patients with COVID-19 that had been cared for by responding organisations was relatively modest, most organisations had nonetheless made substantial changes to their volunteer deployment because of the prevailing pandemic situation. 47.7% of responding organisations indicated they had changed how they deployed volunteers since the start of the pandemic (21.0% said no change, 31.3% missing data). One hundred and nineteen (39.1%) said they were deploying volunteers less, but only 27 (8.9%) said they were using volunteers more (92, 30.3% missing data). Organisations in Europe were less likely to change volunteer deployment than those from the rest of the world (*p* = 0.04706). Prior to the pandemic, the mean number of estimated volunteers actively deployed within responding organisations was 203.1, but at the time of answering the survey this mean had dropped to 33.1. In Table 3 the change in the type of role the volunteers were and are now fulfilling is displayed.

**Table 3.** Deployment of volunteers in different roles before and during COVID-19.

Roles	Organisations reporting volunteers in these roles pre-pandemic	Organisations reporting volunteers in these roles during their perceived peak of COVID-19 to date	Change
	*n* (%)	*n* (%)	
Direct patient/family facing support	186 (61.2)	54 (17.8)	70.7% decrease
Indirect patient/family facing support (e.g. reception, refreshments, driving)	137 (45.1)	39 (12.8)	71.5% decrease
Back office functions (e.g. finance, gardening etc.)	116 (38.2)	19 (6.3)	83.6% decrease
Fundraising functions (e.g. shops, lottery etc.)	109 (35.9)	10 (3.3)	90.8% decrease
Other roles	47 (15.5)	42 (13.8)	10.6% decrease

Organisations generally reported reductions in patient facing work, and a shift to roles such as home-based administration or delivering items to patients and families:

*We had to pause volunteering, then cancel two types of roles altogether (support visitor, [. . .] room attendant) as they were patient facing. We have only kept or continued indirect volunteers. Additionally, we have had to pause them for stretches when we have had 2nd and 3rd (current) waves. (Respondent 137, North America, Adult, In-patient setting)*

Generally, a shift can also be seen towards the volunteers who are deployed being younger than pre-pandemic, with an increase in the proportion of those estimated to be under 50 years, and a commensurate drop in those over 70 years old (Table 4).

**Table 4.** Estimated proportion of volunteers in each age range pre-COVID-19 and during the COVID-19 pandemic.

Age range of volunteers (in years)	Mean estimated % in this age range pre-COVID-19	Mean estimated % in this age range currently, during the COVID-19 pandemic
⩽18	1.3	1.4
19–30	8.0	11.8
31–50	19.1	30.5
51–70	49.1	45.1
71–80	17.8	9.0
80+	3.9	1.4

Organisations indicated that the perceived increased risk of some of their volunteers, should they contract COVID-19, were seen as a barrier to volunteering during the pandemic:

*The volunteers have been very scared of COVID-19, they are old and some are at risk. We now experience, where we can meet, that it is difficult for many to get started again. Several have used the shutdown as an opportunity to stop volunteering. (Respondent 27, Northern Europe, Adult/Child, multiple settings)*

Organisations that indicated that they were deploying volunteers less or much less were asked to rank a selection of reasons why they had done this, displayed in Table 5. The most common reasons were organisational policies, volunteer vulnerabilities and external regulations.

**Table 5.** Ranking of reasons for deploying volunteers less or much less.

Importance	Reason for reduction in using volunteers	Mean ranking scores
1	Our organisation made a policy decision to stop or reduce use of volunteers during the COVID-19 pandemic.	2.8
2	Our volunteers are mostly considered vulnerable to COVID-19 (e.g. due to age or pre-existing conditions)	3.2
3	The areas that our volunteers were deployed in were stopped because of external regulations or lockdowns (e.g. retail/fundraising)	3.3
4	Our volunteers indicated that they preferred not to volunteer at this time due to fears about COVID-19	3.6
5	National policies or procedures prevented us from deploying volunteers.	4.4
6	Volunteers were no longer available (e.g. they had to provide care for family members, were essential workers elsewhere).	5.5
7	Our organisation did not have the resources to coordinate or support volunteers during the COVID-19 pandemic.	5.6
8	Other	7.7

The free text comments primarily illuminated the reduction in volunteers either due to policy changes, or because the volunteers themselves (or their families) were concerned about the risks:

*Volunteers were stopped from working too soon, deeply missed. When level 4 lockdown ended our 65 and under returned immediately to our IPU [in-patient unit], very soon after others returned to the community as they wished, all at their own discretion. Families were concerned for their loved ones, the measures we put in place from an infection control and return to work perspective reduced worries greatly. (Respondent 221, Australasia, Adult/Child, multiple settings)*

Where volunteers were not deployed during the COVID-19 pandemic, organisations worked to keep contact with their volunteers using telephone (59.5%), email (53.6%), post (29.6%), and via meetings (including online meetings) (36.8%).

## Changes in volunteer training

The amount of training provided to volunteers decreased during the COVID-19 pandemic, with a shift where present to online training, with real time training via video conferencing software used more than asynchronous e-learning (Table 6).

**Table 6.** Volunteer training pre and during the COVID-19 pandemic.

Volunteer training offered	Pre-COVID-19 N (%)	During COVID-19 N (%)
Yes	185 (60.9)	148 (48.7)
No	31 (10.2)	65 (21.4)
Mode of training		
Regular in-person training	148 (48.7)	42 (13.8)
Real-time online training (with the usage of web-based communication software e.g. Zoom)	40 (13.2)	112 (36.8)
E-learning with usage materials available online	36 (11.8)	49 (16.1)
Individual training	77 (25.3)	36 (11.8)
Other	15 (4.9)	10 (3.3)
Specific training offered during COVID-19		
Education on COVID-19		120 (39.5)
Education on infection prevention and control measures		141 (46.4)
Training on use of personal protective equipment		125 (41.1)
Training on COVID-Marshalling (e.g. training to guide people around your organisation, check that PPE is being worn correctly and other infection control measures are being followed).		43 (14.1)
Other		57 (18.8)

## New or changed volunteer roles

Participating organisations were asked if they had created new volunteering roles or ways of volunteering during the COVID-19 pandemic. Only 51 organisations (16.8%) indicated that they had done so, however 108 organisations (35.5%) said they had used (or continued to use) virtual volunteering. Such virtual volunteering was mostly commonly telephone contact between volunteers and patients/family members (83, 27.3%), video calls (67, 22.0%), or text contacts (46, 15.1%). Such contact was also used for bereavement support with 59 (19.4%) using telephone contact and 35 (11.5%) using texts. Virtual volunteering roles were more likely to be created by charitable/non-profit organisations (*p* = 0.00209). New volunteering roles were more likely to be created by private organisations (*p* = 0.00987), or where they had cared for patients with confirmed (by test) cases of COVID-19 (*p* = 0.00113). Table 7 displays the likelihood of organisations providing supportive interventions for volunteers or creating new roles dependent on their experiences of caring for those with COVID-19, or having staff or volunteers with COVID-19. Full details of this analysis are found in Supplemental Materials (S3).

**Table 7.** Relationships between organisational experience of COVID-19 and approaches to volunteering.

Organisations that cared for patients with suspected (untested but with clinical diagnosis/symptoms) of COVID-19	more often	provided informal/formal support programmes such as debriefing and counselling for staff	than organisations that did not have such experience	*p* = 0.00192
		created new volunteering roles or ways of volunteering during the COVID-19 pandemic		*p* = 0.01920
		created new volunteering COVID-19 specific roles		*p* = 0.00004
Organisations that had the staff with suspected/confirmed COVID-19		provided informal/formal support programmes such as debriefing and counselling for staff		*p* = 0.00221
		created new volunteering roles or ways of volunteering during the COVID-19 pandemic		*p* = 0.02598
Organisations that had physically present volunteers with suspected or confirmed COVID-19		used virtual volunteering		*p* = 0.03163
		created new COVID-19 specific roles for volunteers		*p* = 0.00000
Organisations that had volunteers with suspected or confirmed COVID-19		offered training to volunteers during the COVID-19 pandemic		*p* = 0.04368
		created new volunteering roles or ways of volunteering during the COVID-19 pandemic		*p* = 0.00815

Changed ways of working for some volunteers included support for patients and families (including virtual support, transport, deliveries of groceries), organisational support (including remote administrative and fundraising roles, gardening or kitchen roles), some COVID-19 specific roles (such as delivering PPE, or managing access or lateral flow testing).

*Many services which were previously face-to-face only were provided by telephone or video-conferencing. We developed a new role providing listening support for those who are bereaved, and a team of compassionate neighbours - both of these have started on the phone or through video-conferencing. Some compassionate neighbours are supporting their nominee by letter-writing. We asked some of our patient transport team to help us by collecting and moving retail donations. We have restructured some teams to enable us to meet infection control requirements - e.g. by having volunteers in our cafe to take orders from visitors, serve orders at the table, and clear up and clean when visitors have left. (Respondent 270, UK, Adults, mixed settings)*

## Impact of reduced volunteering

### Impact on care provision

The general overall reduction in volunteer deployment was keenly felt, with 154 (50.6%) of responding organisations saying that it had an impact on their organisation and/or the care of patients and families, and only 51 (16.8%) of respondents indicating that it had not had an impact. Organisations identified impact on patients and families, on staff, and on the organisations themselves. For patients they perceived reduced support, and increased isolation and loneliness, affecting the patient experience:

*Terrible, a lot of patients and families did not have the support they needed. In a clinic for example even if we have a signed contract with them to visit patient they banned all the visit since first lockdown and still now. . . (Respondent 30, Western Europe, Adult, specialist palliative home care service)*

*People remained alone with their grief, are lonely, had little or no social contact, had to die alone. (Respondent 39, Western Europe, Adult/Child, mixed settings)*

### Impact on the organisation

Lack of volunteer involvement meant less support for staff, increased staff pressure and workload as staff tried to compensate by taking on the roles that volunteers had previously fulfilled:

*Has put additional pressure on paid staff who have to cover roles previously filled by volunteers. (Respondent 115, Western Europe, Adult/Child, mixed settings)*

*Very often our volunteers are seen as equally necessary in caring for our patients. They help our nurses with washing patients, give and prepare food, making beds,. . . when there are no volunteers nurses can’t take care of as many patients at the same time because they are understaffed. (Respondent 239, Western Europe, Adults, In-patient setting)*

Organisations also noted a poorer quality of service, and a different atmosphere without the joy, fun and ‘normality’ that volunteers bring.

*There has been a significant impact on the atmosphere in each hospice setting. The role volunteers play in enabling conversation and joy has been deeply missed. (Respondent 286, UK, Adult, mixed settings)*

*Volunteers made our space more lively and caring for patients and their families. The patients don’t notice the impact but we do. We know that volunteers can help stave off loneliness in patients who have no care circle, and can fill in the voids when family/friends aren’t able to visit. (Respondent 117, Western Europe, Adult, mixed settings)*

## Discussion

### Main findings

The high reduction in the deployment of volunteers in specialist palliative care organisations across the world appears to be sustained over a year into the COVID-19 pandemic. The most common reasons given for this sustained reduction was because of the organisations own policy decision to do so, the vulnerability of current volunteers, or the impact of external regulations/lockdowns. A shift was noted to volunteers being generally younger. However, few organisations had created new volunteer roles or ways of working. Over half of organisations responding perceived that this reduction in volunteers had affected care quality.

### What this study adds

Volunteers are known to contribute to safe and effective palliative care, and enhance patient satisfaction.^20,21^ It is likely that much of the impact of volunteers is in enabling social relationships, ‘being with’ patients, and providing social support.^22,23^ This contribution is impactful, known to have a substantial effect on health and wellbeing.^24,25^ The major reductions in the deployment of volunteers found and sustained thus far through the COVID-19 pandemic must therefore be recognised as likely to have a large impact on care and care outcomes. Volunteers also contribute to the sustainability of specialist palliative care organisations, supporting important functions such as fundraising and income generation, as well as supplementing paid staff in office functions.^9,10^ Organisations must recognise the impact of this deficit, and see volunteers as an essential component of the organisation, not purely an added extra. If interventions are not put in place to enable the return of volunteers to specialist palliative care organisations then it is likely that there will be adverse outcomes at both personal and organisational levels.

The COVID-19 pandemic appears to have accelerated already anticipated changes in patterns and types of volunteering. This includes trends for a more episodic styles of volunteering,^26^ including so-called ‘micro-volunteering’.^27^ Such changes are likely to challenge specialist palliative care volunteering programmes that have typically have relied on ‘constant’ volunteers, rather than those who are ‘serial’ volunteers, or responding to need as a ‘trigger’ volunteer.^28^ It is imperative that urgent attention is given to addressing these changes as despite stated desires to return to previous volunteering patterns,^29^ it is unlikely that this will be fully possible. Specialist palliative care organisations must give attention to how they attract, recruit, train, and construct meaningful roles for volunteers, including those that are virtual or remote, for those who may have different amounts of time to give in unexpected or different patterns.

The policy response of most organisations to restrict or reduce the deployment of volunteers within their organisation stands in stark contrast to rise of volunteering in general during the COVID-19 pandemic. Social action and neighbourhood volunteering were common pandemic responses, with social networks, local knowledge and social trust associated with community organising and volunteering.^30,31^ Place and identity are important determinants of volunteering, with meaning ascribed to the relationship between people and their localities.^32^ There has not been sufficiently strong engagement between such ‘ground up’, locality-based volunteering opportunities and public institutions during the COVID-19 pandemic.^30,31^ Whilst impressive in responsiveness and scale, such social action or neighbourhood volunteering initiatives are not a panacea; volunteers were not equally distributed across communities and were mostly women, middle-class, highly educated and of working age.^31^ Underlying social inequalities are known to present substantive barriers to volunteering.^33^ Specialist palliative care organisations should act to bridge these worlds, building on the strengths of both to build a responsive offer that also has the potential to be attuned to promoting equity in volunteering opportunities. There are existing examples of initiatives acting in such a responsive manner both pre and during COVID-19,^34,35^ and strong voices calling for such community involvement and ownership.^36^ However, there is currently a disjunct for many between the relative formality of their volunteering programmes and the flexibility and responsiveness of community-based initiatives. It has been argued that in order to enable and sustain resilient and confident, ‘disaster-proof’ communities, areas which merit attention include how to engage and support active citizens, new (digital) ways of engagement, transforming formal organisations, and alignment with the (local) context.^37^ If hospice and palliative care organisations are to thrive in a pandemic (and hopefully post-pandemic) world they must seize this opportunity to consider the future role and function of volunteers, considering how to offer more flexible, innovative opportunities rooted in place and locality.

The contributions of volunteers remain relatively under-researched, and this survey has pointed to a number of potential areas for future research: exploring the role and contribution of a new cadre of younger volunteers offering different skills and patterns of availability; understanding in more depth and detail the personal and organisational relationships between volunteers, staff and organisations; and detailed exploration of the possibilities and limitations of virtual and remote volunteering in the specific area of specialist palliative care.

### Strengths and limitations

This was a large, multi-national survey with closed and free-text design giving insight and understanding. However, the pattern of responses is geographically clustered (e.g. many respondents from Germany, Italy and the UK), and this may have affected the results in unknown ways, and it was not possible to analyse per country because of small numbers from most countries. There are major cultural and linguistic differences across participants and this may have affected the interpretation of questions, and hence the response given. The survey was completed by volunteer leads, and hence reflects their views, not those of volunteers themselves. Free text comments, whilst commonly given, were often short with little context, in answer to set questions, so it was not always possible to fully interpret justifications for decisions made and the questions posed may have influenced the breadth of answers given.

## Conclusion

The continued major reduction in the previously common deployment of volunteers within specialist palliative care services is likely to have a continuing negative effect on care provision. It is imperative that services find ways to creatively deploy volunteers in ways that mitigate risk, but offer flexible and responsive volunteering opportunities matched to the skills and availability present in the communities they serve.

## Supplemental Material

sj-pdf-1-pmj-10.1177_02692163221135349 – Supplemental material for Understanding the role and deployment of volunteers within specialist palliative care services and organisations as they have adjusted to the COVID-19 pandemic: A multi-national EAPC volunteer taskforce survey Click here for additional data file. Supplemental material, sj-pdf-1-pmj-10.1177_02692163221135349 for Understanding the role and deployment of volunteers within specialist palliative care services and organisations as they have adjusted to the COVID-19 pandemic: A multi-national EAPC volunteer taskforce survey by Catherine Walshe, Leszek Pawłowski, Sophie Shedel, Steven Vanderstichelen, Melissa J Bloomer, Anne Goossensen, Joaquín T Limonero, Karen Sangild Stoelen, Chiara Caraffa, Leena Pelttari and Ros Scott in Palliative Medicine
