# Supplementary material for: Understanding the role and deployment of volunteers within specialist palliative care services and organisations as they have adjusted to the COVID-19 pandemic: A multi-national EAPC volunteer taskforce survey
Source: Palliat Med. 2023 Feb;37(2):203–14. doi: 10.1177/02692163221135349 (PMC9705505; doi:10.1177/02692163221135349)
Supplement: sj-pdf-1-pmj-10.1177_02692163221135349 – Supplemental material for Understanding the role and deployment of volunteers within specialist palliative care services and organisations as they have adjusted to the COVID-19 pandemic: A multi-national EAPC volunteer taskforce survey [file sj-pdf-1-pmj-10.1177_02692163221135349.pdf]

**Supplementary materials 1. Survey Text (survey itself was delivered via Qualtrics)**

**Understanding the contribution of volunteers to hospice and specialist palliative care services and organisations during the COVID-19 pandemic. An international survey.**

Thank you for your interest in completing this survey. We are trying to find out how the deployment of volunteers in hospice and specialist palliative care organisations and services has changed, or might develop in the future, as a result of the COVID-19 pandemic. This is important as the disease is new and hospices/palliative care organisations and services are changing how they work and there is an opportunity to learn from each other. We are doing this work as part of the EAPC Volunteer Task force.

We realise you are very busy right now, and so we have tried to balance collecting the information that volunteers, patients, policy makers and services think is most helpful, with keeping the questionnaire as short as we can. The survey has 6 sections, and should take no longer than 30 minutes to complete, although it may depend on how much additional/open comments you wish to share.

We ask that this survey is only completed once for each organisation, ideally by the person who has responsibility for managing or organising volunteers within the organisation. By organisation we mean an entity (that could be publicly or privately funded) that provides hospice or specialist palliative care. This may be across one or a number of services (e.g. in-patient care, day care, care at home or in the community). If we think that more than one person from an organisation has completed the survey, we will contact you to clarify if this is the case and how you want us to use your data.

We will consider everything that you say. Your reply will help us. We will share the results of this survey through publications and presentations. The results will be aggregated and anonymised so no-one should be able to tell which organisation has provided particular information. We do not think there are particular risks to completing this survey. Completion of this survey implies consent for your data to be used as part of this study. You will input your data into a secure online survey platform, and these data will be then stored in a secure institutional filestore at Lancaster University.

If you wish to speak to anyone about this survey you can contact the principal investigator Professor Catherine Walshe (c.walshe@lancaster.ac.uk). You may also contact her if you wish to withdraw your responses, up to 2 weeks following completion. If you have made a partial response we may contact you after a week to check if this is an error.

This study has been granted research ethics approval from Lancaster University Faculty of Health and Medicine Research Ethics Committee (FHMREC reference FHMREC20131). If you wish to speak with someone independently about this research you can contact the Director of Research Professor Fiona Lobban (f.lobban@lancaster.ac.uk).

If you are happy to proceed, please consent to participate by clicking below which will take you to the first page of the survey. [CLICK HERE]

SCREENING QUESTIONS:

| | |
|---|---|
| Are you answering on behalf of a palliative or hospice care organisation (this can include: hospices, hospital based palliative care teams/wards, home care/community teams and other services that offer specialist palliative and/or end of life care)? If your organisation offers or coordinates several of these services please complete this survey only once for the organisation as a whole. | Yes<br><br>No<br><br>IF NO then survey finishes |
| Are you responsible for managing volunteers within your organisation, or can provide answers about the use and deployment of volunteers within your organisation? You may be a paid member of staff or a volunteer. | Yes<br><br>No<br><br>If NO then survey finishes |

INFORMATION ABOUT THE PERSON COMPLETING THE SURVEY

This information will only be used in case we need to check back with you, for example if the survey doesn't save correctly. This information will be stored separately to the data that you provide about volunteers and your organisation, to adhere to GDPR and maintain confidentiality and anonymity.

| | |
|---|---|
| What is your name? | Free text |
| What is your contact email address? | Free text |
| What organisation are you answering on behalf of? Please insert name of your organisation here. | Free text |

ORGANISATION INFORMATION

| Question | Response options |
|---|---|
| Date of completion of survey | DD MM YYYY |
| Country | Free text and/or drop-down list of country |
| Are you responsible for coordinating or managing the volunteers within your organisation? | Y/N<br><br>If N what is your role, or in what capacity are you completing this survey? |

| | |
|---|---|
| Does your organisation provide care to only adults, only children, or both? | Adults/Children/Both |
| In what settings do you provide specialist palliative care? (Please answer this with reference to your typical care settings pre-pandemic). Tick all that apply. | In-patient hospice/ward/palliative care unit<br><br>Palliative day care centres/services<br><br>Hospital palliative care advisory team<br><br>Specialist palliative home care service (supporting or consulting about patients at home and/or in the community)<br><br>Providing hands on nursing care at home/in the community (e.g. hospice @ home, pall@home)<br><br>Tick all that apply |
| Do you offer bereavement services? | Yes/No<br><br>If yes, what services are usually provided and to whom? (Free text)<br><br>Did you offer bereavement services only to families/friends of patients who had been cared for by your service? Yes/No |
| How is your organisation primarily managed? Indicate the main source of funding for your organisation. | Charitable / non-profit<br><br>Public<br><br>Private<br><br>Other (a box will open below) |
| | |

EXPERIENCE WITH COVID-19

| | |
|---|---|
| Have your services within your organisation cared for patients with confirmed (by test) cases of COVID-19? | Yes/No<br><br>If yes, approximately how many confirmed cases since January 2020. |

| | |
|---|---|
| Have your services within your organisation cared for patients with suspected (untested but with clinical diagnosis/symptoms) of COVID-19? | Yes/No<br><br>If yes, approximately how many suspected cases since January 2020. |
| Have your services within your organisation had staff with suspected/confirmed COVID-19? | Yes/No<br><br>If yes, approximately how many staff have had suspected or confirmed COVID-19 since January 2020? |
| Have your services within your organisation had volunteers with suspected or confirmed COVID-19? | Yes/No<br><br>If yes, approximately how many volunteers have had suspected or confirmed COVID-19 since January 2020? Free text number.<br><br>If yes, we would like to understand a little more about whether you have concerns that volunteers were infected with COVID-19 because of their association with your organisation, or if you think community transmission was more likely. We understand that you will not know the actual route of transmission.<br><br>Had volunteers with suspected or confirmed COVID-19 been physically present in your organisation/services such that their association with your organisation might have been a route of transmission?<br><br>Yes/No |

| | |
|---|---|
| When do you estimate the peak of COVID to have occurred for your organisation? By peak of COVID we mean the point at which you were caring for the highest number of people with a COVID diagnosis and/or the numbers of those with COVID in the communities you serve were the highest. | MM/YY |
| Is there anything about your organisations experience with COVID related to the deployment of volunteers that you want to tell us? | Free text |

VOLUNTEERS WITHIN YOUR ORGANISATION

| | |
|---|---|
| Did your organisation have volunteer roles available pre-pandemic? | Yes/No |
| IF YES | |
| Prior to COVID-19, how many volunteers in total were active within your service/organisation. We appreciate this may be an estimation. By active we mean providing volunteer hours on a regular basis to the organisation e.g. at least monthly | Numerical answer |
| Prior to COVID-19 what roles did volunteers have? Tick all that apply | Direct patient/family facing support<br>Indirect patient / family facing support (e.g. reception functions, refreshments, driving / transport etc.)<br>Back office functions (e.g. finance support, maintenance, gardening etc.)<br>Fundraising functions (e.g. shop volunteers, lottery etc.)<br>Others (a box will open below) |

| | |
|---|---|
| Prior to COVID-19 what were the typical age ranges of active volunteers within your organisation. We appreciate you may not know the exact ages of your volunteers. We would like you to estimate what proportion of your volunteers fit into each of these age categories (total should add to 100%). | Age range Proportion<br><br>≤18<br><br>19-30<br><br>31-50<br><br>51-70<br><br>71-80<br><br>80+ |
| Have you changed how you deploy volunteers since COVID-19? | Yes/No<br><br><br>Please give details. |
| How would you say you are deploying volunteers now compared to before the pandemic? Please answer to give the position at the date of answering the survey – this may have fluctuated since the start of the pandemic. | A lot more<br><br>Slightly more<br><br>About the same<br><br>Slightly less<br><br>Much less |
| If you are deploying volunteers less or much less please let us know why this is? Please order from the most important to the least important or not applicable to your organisation. | Our volunteers are mostly considered vulnerable to COVID-19 (e.g. due to age or pre-existing conditions)<br><br><br>Our volunteers indicated that they preferred not to volunteer at this time due to fears about COVID-19<br><br><br>Our organisation made a policy decision to stop or reduce use of volunteers during the COVID-19 pandemic. |

| | |
|---|---|
| | Our organisation did not have the resources to coordinate or support volunteers during the COVID-19 pandemic.<br><br>The areas that our volunteers were deployed in were stopped because of external regulations or lockdowns (e.g. retail/fundraising)<br><br>Volunteers were no longer available (e.g. they had to provide care for family members, were essential workers elsewhere).<br><br>National policies or procedures prevented us from deploying volunteers.<br><br>Other |
| Do you think that the changes in volunteer deployment have had an impact on your organisation or the care of patients/families? | Yes/No<br><br>If yes, what has this impact been? Free text. |
| At this point in time how many volunteers are active in your service/organisation? We appreciate this may be an estimation. By active we mean providing volunteer hours on a regular basis to the organisation e.g. at least monthly. | Numerical answer |
| IF NO to question about deployment of volunteers pre-pandemic | |
| Have you deployed volunteers since the COVID-19 pandemic began? | Yes/No |
| IF YES to above question | |
| What roles have you deployed volunteers in to? Please tick all that apply. | Direct patient/family facing support |

| | |
|---|---|
| | Indirect patient / family facing support (e.g. reception functions, refreshments, driving / transport etc.)<br>Back office functions (e.g. finance support, maintenance, gardening etc.)<br>Fundraising functions (e.g. shop volunteers, lottery etc.)<br>Others (a box will open below) |
| At this point in time (date of answering this survey) how many volunteers are active in your service/organisation? We appreciate this may be an estimation. By active we mean providing volunteer hours on a regular basis to the organisation e.g. at least monthly | Numerical answer |
| | |
| How many volunteers were active within your service/organisation at the PEAK of COVID-19 that you have experienced thus far? We appreciate this may be an estimation. By active we mean providing volunteer hours on a regular basis to the organisation e.g. at least monthly | Numerical answer |
| In what services were volunteers primarily deployed into (if any) at the PEAK of COVID-19. Please tick one box only to indicate the main deployment of volunteers at the peak of COVID-19 | No volunteers deployed<br>Direct patient/family facing support<br>Indirect patient / family facing support (e.g. reception functions, refreshments, driving / transport etc.)<br>Back office functions (e.g. finance support, maintenance, gardening etc.)<br>Fundraising functions (e.g. shop volunteers, lottery etc.) |

| | |
|---|---|
| | Others (a box will open below) |
| Currently, during the COVID-19 pandemic what were the typical age ranges of active volunteers within your organisation. We appreciate you may not know the exact ages of your volunteers. We would like you to estimate what proportion of your volunteers fit into each of these age categories (total should add to 100%). | Age range Proportion<br>≤18<br>19-30<br>31-50<br>51-70<br>71-80<br>80+ |

VOLUNTEER TRAINING

| | |
|---|---|
| Did you offer training for volunteers pre-COVID-19? | Yes/No |
| IF YES | |
| How did you offer training to volunteers pre-COVID-19? Please tick all that apply. | Regular in-person training<br>Real-time online training (with the usage of web-based communication software e.g. Zoom)<br>E-learning with usage materials available online<br>Individual training<br>Other (please specify) |
| Have you offered training to volunteers during the COVID-19 pandemic? | Yes/No |
| IF YES | |
| How have you offered training to volunteers during the COVID-19 pandemic? Please tick all that apply. | Regular in-person training<br>Real-time online training (with the usage of web-based communication software e.g. Zoom)<br>E-learning with usage materials available online<br>Individual training<br>Other (please specify) |

| | |
|---|---|
| Where volunteers continue to attend your service/organisation to fulfil a role, were they provided with the following? – tick all that apply | Education on COVID-19<br><br>Education on infection prevention and control measures<br><br>Training on use of personal protective equipment<br><br>Training on COVID-Marshalling (e.g. training to guide people around your organisation, check that PPE is being worn correctly and other infection control measures are being followed).<br><br>Other (please specify) |

CARE AND SUPPORT FOR VOLUNTEERS DURING COVID-19

| | |
|---|---|
| Have you been able to facilitate COVID-19 vaccination for people within the organisation? | Yes/No |
| IF YES | |
| Were volunteers able to access the vaccination programme that your organisation was able to facilitate. | Yes/No |
| IF YES | |
| Were all volunteers given access to this vaccination programme | Yes/No<br><br>If no please explain why not, or which criteria were used to prioritise vaccinations. |
| Have you kept contact with volunteers who have been inactive during COVID-19 | Yes/No |
| IF YES | |
| How have you kept contact with volunteers? | Telephone<br><br>Email |

| | |
|---|---|
| | Postal contact e.g. letters or newsletters<br><br>Regular meetings (including via video-or-telephone conferencing)<br><br>Other<br><br>Tick all that apply |
| What have you done to try and retain your volunteers during COVID-19<br><br>How will you enable the engagement or return of volunteers post COVID-19? | Free text answer |
| Since COVID-19, does your service/organisation provide informal/formal support programs such as debriefing and counselling for staff?<br><br>a. If so, are these services also offered to volunteers? | Yes/No |

NEW VOLUNTEERING ROLES DURING COVID-19 OR FOR THE FUTURE

| | |
|---|---|
| Have you created new volunteering roles or ways of volunteering during the COVID-19 pandemic? | Yes/No |
| IF YES | |
| What new volunteering roles or ways of volunteering have you created? | Free text |
| Have you used virtual volunteering roles at all? If so what sort of virtual volunteering have you used. Tick all that apply. | Telephone contact between volunteers and patients/family members.<br><br>Video call (e.g. Zoom/Teams) contact between volunteers and patients/family members. |

| | |
|---|---|
| | WhatsApp or text contact between volunteers and patients/family members.<br><br>Telephone contact for bereavement care services.<br><br>Video call contact for bereavement care services.<br><br>WhatsApp or text/messaging contact for bereavement care services.<br><br>Off-site support roles (e.g. sewing scrubs, making support packs up, administration off site)<br><br>Other (please specify) |
| Have you created new roles for volunteers that are COVID-19 specific. | Yes/No<br><br>COVID-19 marshalling (e.g. guiding people around your organisation, checking that PPE is being worn correctly and other infection control measures are being followed).<br><br>Maintaining stocks of personal protective equipment and other relevant equipment<br><br>Facilitating COVID-19 secure family visiting (e.g. window visits).<br><br>Visitor screening (e.g. temperature checking, issuing personal protective equipment, enabling COVID-19 testing).<br><br>Other. |

| | |
|---|---|
| Do you have plans for volunteers for the future? What are these plans, and what do you think will help or hinder these? | Free text |
| | |
| Once the pandemic is over do you plan to | bring volunteers back into the same roles as before<br><br>enable volunteers to work in the same way as before<br><br>change the way volunteers work ( please give details)<br><br>Check all that apply. |
| Is there anything else about volunteers in your organisation at this time of COVID that you think it is important that we know? Please tell us what this is. | Free text. |

**Supplementary material 2: Number of responding organisations by country**

| Responding organisations by country | | |
|---|---|---|
| | n | % |
| Argentina | 4 | 1.31 |
| Australia | 13 | 4.28 |
| Austria | 8 | 2.63 |
| Belgium | 14 | 4.61 |
| Canada | 4 | 1.32 |
| Chile | 1 | 0.33 |
| Denmark | 10 | 3.29 |
| Finland | 2 | 0.66 |
| France | 8 | 2.63 |
| Germany | 77 | 25.33 |
| Greece | 1 | 0.33 |
| Hungary | 3 | 0.99 |
| India | 10 | 3.29 |
| Iran | 1 | 0.33 |
| Ireland | 4 | 1.32 |
| Italy | 43 | 14.14 |
| Kenya | 1 | 0.33 |
| Lithuania | 1 | 0.33 |
| New Zealand | 1 | 0.33 |
| The Netherlands | 4 | 1.32 |
| Norway | 3 | 0.99 |
| Poland | 5 | 1.64 |
| Republic of Moldova | 1 | 0.33 |

| | | |
|---|---|---|
| Romania | 1 | 0.33 |
| Russia | 1 | 0.33 |
| Rwanda | 1 | 0.33 |
| Serbia | 1 | 0.33 |
| Singapore | 3 | 0.99 |
| Slovakia | 1 | 0.33 |
| Spain | 5 | 1.64 |
| Sweden | 2 | 0.66 |
| Switzerland | 2 | 0.66 |
| UK | 46 | 15.13 |
| Ukraine | 2 | 0.66 |
| The United States | 20 | 6.58 |

**Supplementary material 3. Full results of statistical analysis.**

1.

| Had volunteers with suspected or confirmed COVID-19 been physically present in your organisation/services such that their association with your organisation might have been a route of transmission? | Have you created new roles for volunteers that are COVID-19 specific? Yes | Have you created new roles for volunteers that are COVID-19 specific? No | Total in rows |
|---|---|---|---|
| | | | |
| Yes | 15 | 7 | 22 |
| % of the column | 55.56% | 10.29% | |
| % of the row | 68.18% | 31.82% | |
| No | 12 | 61 | 73 |
| % of the column | 44.44% | 89.71% | |
| % of the row | 16.44% | 83.56% | |
| Total | 27 | 68 | 95 |

| | Chi-square | df | p |
|---|---|---|---|
| The Pearson Chi-square test | 22.24884 | df=1 | p=.00000 |
| The Maximum-Likelihood Chi-square test | 20.64404 | df=1 | p=.00001 |

Organisations that had  physically present volunteers with suspected or confirmed COVID-19. more often created the new COVID-19 specific roles for volunteers in comparison to organisations which had healthy volunteers  (p=0.00000)

2.

| Had volunteers with suspected or confirmed COVID-19 been physically present in your organisation/services such that their association with your organisation might have been a route of transmission? | Have you used virtual volunteering roles at all? Yes | Have you used virtual volunteering roles at all? No | Total in rows |
|---|---|---|---|
| Yes | 17 | 5 | 22 |
| % of the column | 31.48% | 12.50% | |
| % of the row | 77.27% | 22.73% | |
| No | 37 | 35 | 72 |
| % of the column | 68.52% | 87.50% | |
| % of the row | 51.39% | 48.61% | |
| Total | 54 | 40 | 94 |

| | | | |
|---|---|---|---|
| | Chi-square | df | p |
| The Pearson Chi-square test | 4.618348 | df=1 | p=.03163 |
| The Maximum-Likelihood Chi-square test | 4.878919 | df=1 | p=.02719 |

Organisations that had physically present volunteers with suspected or confirmed COVID-19. more often used virtual volunteering roles in comparison to organisations without such experience (p=0.03163).

3.

| Had volunteers with suspected or confirmed COVID-19 been physically present in your organisation/services such that their association with your organisation might have been a route of transmission? | Have you created new volunteering roles or ways of volunteering during the COVID-19 pandemic? Yes | Have you created new volunteering roles or ways of volunteering during the COVID-19 pandemic? No | Total in rows |
|---|---|---|---|
| Yes | 17 | 5 | 22 |
| % of the column | 30.91% | 12.50% | |
| % of the row | 77.27% | 22.73% | |
| No | 38 | 35 | 73 |
| % of the column | 69.09% | 87.50% | |
| % of the row | 52.05% | 47.95% | |
| Total | 55 | 40 | 95 |

| | | | |
|---|---|---|---|
| | Chi-square | df | p |
| The Pearson Chi-square test | 4.410273 | df=1 | p=.03572 |
| The Maximum-Likelihood Chi-square test | 4.661201 | df=1 | p=.03085 |

4.

| Had volunteers with suspected or confirmed COVID-19 been physically present in your organisation/services such that their association with your organisation might have been a route of transmission? | Since COVID-19. does your service/organisation provide informal/formal support programs such as debriefing and counselling for staff? Yes | Since COVID-19. does your service/organisation provide informal/formal support programs such as debriefing and counselling for staff? No | Total in rows |
|---|---|---|---|
| Yes | 21 | 1 | 22 |
| % of the column | 28.00% | 5.26% | |
| % of the row | 95.45% | 4.55% | |
| No | 54 | 18 | 72 |

| | | | |
|---|---|---|---|
| % of the column | 72.00% | 94.74% | |
| % of the row | 75.00% | 25.00% | |
| Total | 75 | 19 | 94 |

| | | | |
|---|---|---|---|
| | Chi-square | df | p |
| The Pearson Chi-square test | 4.371675 | df=1 | p=.03654 |
| The Maximum-Likelihood Chi-square test | 5.515335 | df=1 | p=.01885 |

5.

| Had volunteers with suspected or confirmed COVID-19 been physically present in your organisation/services such that their association with your organisation might have been a route of transmission? | Have you offered training to volunteers during the COVID-19 pandemic? No | Have you offered training to volunteers during the COVID-19 pandemic? Yes | Total in rows |
|---|---|---|---|
| Yes | 1 | 20 | 21 |
| % of the column | 4.17% | 27.03% | |
| % of the row | 4.76% | 95.24% | |
| No | 23 | 54 | 77 |
| % of the column | 95.83% | 72.97% | |
| % of the row | 29.87% | 70.13% | |
| Total | 24 | 74 | 98 |

| | | | |
|---|---|---|---|
| | Chi-square | df | p |
| The Pearson Chi-square test | 5.625034 | df=1 | p=.01771 |
| The Maximum-Likelihood Chi-square test | 7.161733 | df=1 | p=.00745 |

6.

| Had volunteers with suspected or confirmed COVID-19 been physically present in your organisation/s | How would you say you are deploying volunteers now compared to before the pandemic? Please answer to give the position at the date of answering the | How would you say you are deploying volunteers now compared to before the pandemic? Please answer to give the position at the date of answering the | How would you say you are deploying volunteers now compared to before the pandemic? Please answer to give the position at the date of answering the | How would you say you are deploying volunteers now compared to before the pandemic? Please answer to give the position at the date of answering the | How would you say you are deploying volunteers now compared to before the pandemic? Please answer to give the position at the date of answering the | Total in rows |
|---|---|---|---|---|---|---|

| ervices such that their association with your organisation might have been a route of transmission? | survey – this may have fluctuated since the start of the pandemic. Much less | survey – this may have fluctuated since the start of the pandemic. Slightly less | survey – this may have fluctuated since the start of the pandemic. About the same | survey – this may have fluctuated since the start of the pandemic. Slightly more | survey – this may have fluctuated since the start of the pandemic. A lot more | |
|---|---|---|---|---|---|---|
| Yes | 3 | 3 | 7 | 5 | 3 | 21 |
| % of the column | 12.00% | 11.54% | 26.92% | 55.56% | 42.86% | |
| % of the row | 14.29% | 14.29% | 33.33% | 23.81% | 14.29% | |
| No | 22 | 23 | 19 | 4 | 4 | 72 |
| % of the column | 88.00% | 88.46% | 73.08% | 44.44% | 57.14% | |
| % of the row | 30.56% | 31.94% | 26.39% | 5.56% | 5.56% | |
| Total | 25 | 26 | 26 | 9 | 7 | 93 |

| | Chi-square | df | p |
|---|---|---|---|
| The Pearson Chi-square test | 10.93896 | df=4 | p=.02726 |
| The Maximum-Likelihood Chi-square test | 10.19516 | df=4 | p=.03727 |

7.

| Have your services within your organisation had volunteers with suspected or confirmed COVID-19? | Have you created new volunteering roles or ways of volunteering during the COVID-19 pandemic? Yes | Have you created new volunteering roles or ways of volunteering during the COVID-19 pandemic? No | Total in rows |
|---|---|---|---|
| No | 46 | 66 | 112 |
| % of the column | 45.10% | 63.46% | |
| % of the row | 41.07% | 58.93% | |
| Yes | 56 | 38 | 94 |
| % of the column | 54.90% | 36.54% | |
| % of the row | 59.57% | 40.43% | |
| Total | 102 | 104 | 206 |

| | Chi-square | df | p |
|---|---|---|---|

| | | | |
|---|---|---|---|
| The Pearson Chi-square test | 6.999479 | df=1 | p=.00815 |
| The Maximum-Likelihood Chi-square test | 7.039426 | df=1 | p=.00797 |

Organisations that had volunteers with suspected or confirmed COVID-19 more often created new volunteering roles or ways of volunteering during the COVID-19 pandemic than the organisations that did not have such experience (p=0.00815).

8.

| Have your services within your organisation had volunteers with suspected or confirmed COVID-19? | Since COVID-19. does your service/organisation provide informal/formal support programs such as debriefing and counselling for staff? Yes | Since COVID-19. does your service/organisation provide informal/formal support programs such as debriefing and counselling for staff? No | Total in rows |
|---|---|---|---|
| No | 70 | 42 | 112 |
| % of the column | 48.95% | 68.85% | |
| % of the row | 62.50% | 37.50% | |
| Yes | 73 | 19 | 92 |
| % of the column | 51.05% | 31.15% | |
| % of the row | 79.35% | 20.65% | |
| Total | 143 | 61 | 204 |

| | Chi-square | df | p |
|---|---|---|---|
| The Pearson Chi-square test | 6.840028 | df=1 | p=.00891 |
| The Maximum-Likelihood Chi-square test | 6.989278 | df=1 | p=.00820 |

9.

| Have your services within your organisation had volunteers with suspected or confirmed COVID-19? | Have you offered training to volunteers during the COVID-19 pandemic? No | Have you offered training to volunteers during the COVID-19 pandemic? Yes | Total in rows |
|---|---|---|---|
| No | 41 | 71 | 112 |
| % of the column | 64.06% | 48.97% | |
| % of the row | 36.61% | 63.39% | |
| Yes | 23 | 74 | 97 |
| % of the column | 35.94% | 51.03% | |
| % of the row | 23.71% | 76.29% | |
| Total | 64 | 145 | 209 |

| | |
|---|---|
| | |

| | Chi-square | df | p |
|---|---|---|---|
| The Pearson Chi-square test | 4.068973 | df=1 | p=.04368 |
| The Maximum-Likelihood Chi-square test | 4.116040 | df=1 | p=.04248 |

Organisations that had volunteers with suspected or confirmed COVID-19 offered training to volunteers during the COVID-19 pandemic more often that organisations that did not have such experience (p=0.04368).

10.

| Have your services within your organisation had volunteers with suspected or confirmed COVID-19? | Have you changed how you deploy volunteers since COVID-19? Please give details in one of the boxes below. - Selected Choice Yes | Have you changed how you deploy volunteers since COVID-19? Please give details in one of the boxes below. - Selected Choice No | Total in rows |
|---|---|---|---|
| No | 74 | 42 | 116 |
| % of the column | 51.75% | 66.67% | |
| % of the row | 63.79% | 36.21% | |
| Yes | 69 | 21 | 90 |
| % of the column | 48.25% | 33.33% | |
| % of the row | 76.67% | 23.33% | |
| Total | 143 | 63 | 206 |

| | Chi-square | df | p |
|---|---|---|---|
| The Pearson Chi-square test | 3.956295 | df=1 | p=.04670 |
| The Maximum-Likelihood Chi-square test | 4.020298 | df=1 | p=.04496 |

The way of volunteers' deployment since COVID-19 presents more changes in organisations that had volunteers with suspected or confirmed COVID-19 in comparison to organisations which had healthy volunteers (p=0.04670).

11.

| Have your services within your organisation had staff with suspected/confirmed COVID-19? | Have you created new volunteering roles or ways of volunteering during the COVID-19 pandemic? Yes | Have you created new volunteering roles or ways of volunteering during the COVID-19 pandemic? No | Total in rows |
|---|---|---|---|
| Yes | 76 | 62 | 138 |

| | | | |
|---|---|---|---|
| % of the column | 73.08% | 58.49% | |
| % of the row | 55.07% | 44.93% | |
| No | 28 | 44 | 72 |
| % of the column | 26.92% | 41.51% | |
| % of the row | 38.89% | 61.11% | |
| Total | 104 | 106 | 210 |

| | Chi-square | df | p |
|---|---|---|---|
| The Pearson Chi-square test | 4.957247 | df=1 | p=.02598 |
| The Maximum-Likelihood Chi-square test | 4.989101 | df=1 | p=.02551 |

Organisations that had the staff with suspected/confirmed COVID-19 more often created new volunteering roles or ways of volunteering during the COVID-19 pandemic than organisations that did not have such experience (p=0.02598)

12.

| Have your services within your organisation had staff with suspected/confirmed COVID-19? | Since COVID-19. does your service/organisation provide informal/formal support programs such as debriefing and counselling for staff? Yes | Since COVID-19. does your service/organisation provide informal/formal support programs such as debriefing and counselling for staff? No | Total in rows |
|---|---|---|---|
| Yes | 105 | 30 | 135 |
| % of the column | 71.43% | 49.18% | |
| % of the row | 77.78% | 22.22% | |
| No | 42 | 31 | 73 |
| % of the column | 28.57% | 50.82% | |
| % of the row | 57.53% | 42.47% | |
| Total | 147 | 61 | 208 |

| | Chi-square | df | p |
|---|---|---|---|
| The Pearson Chi-square test | 9.367967 | df=1 | p=.00221 |
| The Maximum-Likelihood Chi-square test | 9.145752 | df=1 | p=.00249 |

Organisations that had the staff with suspected/confirmed COVID-19 more often provided informal/formal support programs such as debriefing and counselling for staff than organisations that did not have such experience (p=0.00221)

13.

| Have your services within your organisation cared for patients with suspected (untested but with clinical diagnosis/symptoms) of COVID-19? | Have you created new roles for volunteers that are COVID-19 specific?<br>Yes | Have you created new roles for volunteers that are COVID-19 specific?<br>No | Total in rows |
|---|---|---|---|
| No | 16 | 102 | 118 |
| % of the column | 32.00% | 64.97% | |
| % of the row | 13.56% | 86.44% | |
| Yes | 34 | 55 | 89 |
| % of the column | 68.00% | 35.03% | |
| % of the row | 38.20% | 61.80% | |
| Total | 50 | 157 | 207 |

| | Chi-square | df | p |
|---|---|---|---|
| The Pearson Chi-square test | 16.81734 | df=1 | p=.00004 |
| The Maximum-Likelihood Chi-square test | 16.83971 | df=1 | p=.00004 |

Organisations that cared for patients with suspected (untested but with clinical diagnosis/symptoms) of COVID-19 more often created new volunteering roles that are COVID-19 specific than organisations that did not have such experience (p=0.00004).

14.

| Have your services within your organisation cared for patients with suspected (untested but with clinical diagnosis/symptoms) of COVID-19? | Have you created new volunteering roles or ways of volunteering during the COVID-19 pandemic?<br>Yes | Have you created new volunteering roles or ways of volunteering during the COVID-19 pandemic?<br>No | Total in rows |
|---|---|---|---|
| No | 49 | 68 | 117 |
| % of the column | 48.04% | 64.15% | |
| % of the row | 41.88% | 58.12% | |
| Yes | 53 | 38 | 91 |
| % of the column | 51.96% | 35.85% | |
| % of the row | 58.24% | 41.76% | |
| Total | 102 | 106 | 208 |

| | Chi-square | df | p |
|---|---|---|---|

| | | | |
|---|---|---|---|
| The Pearson Chi-square test | 5.483102 | df=1 | p=.01920 |
| The Maximum-Likelihood Chi-square test | 5.506096 | df=1 | p=.01895 |

Organisations that cared for patients with suspected (untested but with clinical diagnosis/symptoms) of COVID-19 more often created new volunteering roles or ways of volunteering during the COVID-19 pandemic than organisations that did not have such experience (p=0.01920)

15.

| Have your services within your organisation cared for patients with suspected (untested but with clinical diagnosis/symptoms) of COVID-19? | Since COVID-19. does your service/organisation provide informal/formal support programs such as debriefing and counselling for staff? Yes | Since COVID-19. does your service/organisation provide informal/formal support programs such as debriefing and counselling for staff? No | Total in rows |
|---|---|---|---|
| No | 73 | 45 | 118 |
| % of the column | 50.34% | 73.77% | |
| % of the row | 61.86% | 38.14% | |
| Yes | 72 | 16 | 88 |
| % of the column | 49.66% | 26.23% | |
| % of the row | 81.82% | 18.18% | |
| Total | 145 | 61 | 206 |

| | Chi-square | df | p |
|---|---|---|---|
| The Pearson Chi-square test | 9.629066 | df=1 | p=.00192 |
| The Maximum-Likelihood Chi-square test | 9.982197 | df=1 | p=.00158 |

Organisations that cared for patients with suspected (untested but with clinical diagnosis/symptoms) of COVID-19 more often provided informal/formal support programs such as debriefing and counselling for staff than organisations that did not have such expirience (p=0.00192)

16.

| Have your services within your organisation cared for patients with confirmed (by test) cases of COVID-19? | Have you created new roles for volunteers that are COVID-19 specific? Yes | Have you created new roles for volunteers that are COVID-19 specific? No | Total in rows |
|---|---|---|---|
| Yes | 34 | 64 | 98 |
| % of the column | 66.67% | 40.51% | |
| % of the row | 34.69% | 65.31% | |

| | | | |
|---|---|---|---|
| No | 17 | 94 | 111 |
| % of the column | 33.33% | 59.49% | |
| % of the row | 15.32% | 84.68% | |
| Total | 51 | 158 | 209 |

| | Chi-square | df | p |
|---|---|---|---|
| The Pearson Chi-square test | 10.59525 | df=1 | p=.00113 |
| The Maximum-Likelihood Chi-square test | 10.69837 | df=1 | p=.00107 |

Organisations that cared for patients with confirmed (by test) cases of COVID-19 more often created the new COVID-19 specific roles for volunteers in comparison to organisations that did not provide such care (p=0.00113).

17.

| How is your organisation primarily managed? Indicate the main source of funding for your organisation. - Selected Choice | Have you created new roles for volunteers that are COVID-19 specific? Yes | Have you created new roles for volunteers that are COVID-19 specific? No | Total in rows |
|---|---|---|---|
| Charitable / non-profit | 34 | 103 | 137 |
| % of the column | 66.67% | 65.19% | |
| % of the row | 24.82% | 75.18% | |
| Private | 7 | 4 | 11 |
| % of the column | 13.73% | 2.53% | |
| % of the row | 63.64% | 36.36% | |
| Public | 5 | 28 | 33 |
| % of the column | 9.80% | 17.72% | |
| % of the row | 15.15% | 84.85% | |
| Other (please put details in box below) | 5 | 23 | 28 |
| % of the column | 9.80% | 14.56% | |
| % of the row | 17.86% | 82.14% | |
| Total | 51 | 158 | 209 |

| | Chi-square | df | p |
|---|---|---|---|
| The Pearson Chi-square test | 11.37266 | df=3 | p=.00987 |
| The Maximum-Likelihood Chi-square test | 9.972780 | df=3 | p=.01880 |

Private organisations more often than the others created new COVID-19 specific roles for volunteers (p=0.00987).

18.

| How is your organisation primarily managed? Indicate the main source of funding for your organisation. - Selected Choice | Have you used virtual volunteering roles at all? Yes | Have you used virtual volunteering roles at all? No | Total in rows |
|---|---|---|---|
| Charitable / non-profit | 83 | 54 | 137 |
| % of the column | 76.85% | 53.47% | |
| % of the row | 60.58% | 39.42% | |
| Private | 6 | 5 | 11 |
| % of the column | 5.56% | 4.95% | |
| % of the row | 54.55% | 45.45% | |
| Public | 10 | 23 | 33 |
| % of the column | 9.26% | 22.77% | |
| % of the row | 30.30% | 69.70% | |
| Other (please put details in box below) | 9 | 19 | 28 |
| % of the column | 8.33% | 18.81% | |
| % of the row | 32.14% | 67.86% | |
| Total | 108 | 101 | 209 |

| | Chi-square | df | p |
|---|---|---|---|
| The Pearson Chi-square test | 14.70428 | df=3 | p=.00209 |
| The Maximum-Likelihood Chi-square test | 14.95608 | df=3 | p=.00185 |

Charitable / non-profit organisations more often used virtual volunteering roles than the public organisations (p=0.00209).

19.

| How is your organisation primarily managed? Indicate the main source of funding for your organisation. - Selected Choice | Have you created new volunteering roles or ways of volunteering during the COVID-19 pandemic? Yes | Have you created new volunteering roles or ways of volunteering during the COVID-19 pandemic? No | Total in rows |
|---|---|---|---|
| Charitable / non-profit | 80 | 59 | 139 |
| % of the column | 76.92% | 55.66% | |
| % of the row | 57.55% | 42.45% | |
| Private | 5 | 6 | 11 |
| % of the column | 4.81% | 5.66% | |
| % of the row | 45.45% | 54.55% | |
| Public | 10 | 22 | 32 |
| % of the column | 9.62% | 20.75% | |
| % of the row | 31.25% | 68.75% | |
| Other (please put details in box below) | 9 | 19 | 28 |

| | | | |
|---|---|---|---|
| % of the column | 8.65% | 17.92% | |
| % of the row | 32.14% | 67.86% | |
| Total | 104 | 106 | 210 |

| | | | |
|---|---|---|---|
| | Chi-square | df | p |
| The Pearson Chi-square test | 11.31698 | df=3 | p=.01013 |
| The Maximum-Likelihood Chi-square test | 11.52023 | df=3 | p=.00922 |

20.

| Q8a (1-Europe. 2-rest of the World) | Have you changed how you deploy volunteers since COVID-19? Please give details in one of the boxes below. - Selected Choice Yes | Have you changed how you deploy volunteers since COVID-19? Please give details in one of the boxes below. - Selected Choice No | Total in rows |
|---|---|---|---|
| 1 | 105 | 55 | 160 |
| % of the column | 73.43% | 85.94% | |
| % of the row | 65.63% | 34.38% | |
| 2 | 38 | 9 | 47 |
| % of the column | 26.57% | 14.06% | |
| % of the row | 80.85% | 19.15% | |
| Total | 143 | 64 | 207 |

| | | | |
|---|---|---|---|
| | Chi-square | df | p |
| The Pearson Chi-square test | 3.943188 | df=1 | p=.04706 |
| The Maximum-Likelihood Chi-square test | 4.210401 | df=1 | p=.04018 |

The way of volunteers' deployment since COVID-19 presents less changes in European organisations than organisations from the rest of the World. (p=0.04706)

21.

| How would you say you are deploying volunteers now compared to before the pandemic? Please answer to give the position | Had volunteers with suspected or confirmed COVID-19 been physically present in your organisation/services such | Had volunteers with suspected or confirmed COVID-19 been physically present in your organisation/services such | Total in |
|---|---|---|---|

| at the date of answering the survey – this may have fluctuated since the start of the pandemic. More - 1. less-2 | that their association with your organisation might have been a route of transmission? Yes | that their association with your organisation might have been a route of transmission? No | rows |
|---|---|---|---|
| 1 | 8 | 8 | 16 |
| % of the column | 57.14% | 15.09% | |
| % of the row | 50.00% | 50.00% | |
| 2 | 6 | 45 | 51 |
| % of the column | 42.86% | 84.91% | |
| % of the row | 11.76% | 88.24% | |
| Total | 14 | 53 | 67 |

| | Chi-square | df | p |
|---|---|---|---|
| The Pearson Chi-square test | 10.77184 | df=1 | p=.00103 |
| The Maximum-Likelihood Chi-square test | 9.558076 | df=1 | p=.00199 |

22.

| How would you say you are deploying volunteers now compared to before the pandemic? Please answer to give the position at the date of answering the survey – this may have fluctuated since the start of the pandemic. More - 1. less-2 | Q8a (1-Europe. 2-rest of the World) 1 | Q8a (1-Europe. 2-rest of the World) 2 | Total in rows |
|---|---|---|---|
| 1 | 15 | 11 | 26 |
| % of the column | 13.27% | 35.48% | |
| % of the row | 57.69% | 42.31% | |
| 2 | 98 | 20 | 118 |
| % of the column | 86.73% | 64.52% | |
| % of the row | 83.05% | 16.95% | |
| Total | 113 | 31 | 144 |

| | Chi-square | df | p |
|---|---|---|---|
| The Pearson Chi-square test | 8.110100 | df=1 | p=.00440 |
| The Maximum-Likelihood Chi-square test | 7.184904 | df=1 | p=.00735 |

The number of volunteers in the organisations compared to before the pandemic dropped to higher extend in Europe than in organisations from the rest of the World (p=0.00440)
